# Sam68 stimulates polymerase theta-mediated end-joining by suppressing poison exon inclusion in *Polq* mRNA

**DOI:** 10.1093/nar/gkag783

**Published:** 2026-08-11

**Authors:** Marloes D van Wezel, Hanneke Kool, Diana van den Heuvel, Robin van Schendel, Marco Barazas, Martijn S Luijsterburg, Marcel Tijsterman, Joost Schimmel

**Affiliations:** Department of Human Genetics, Leiden University Medical Center, Leiden, 2333 ZA, The Netherlands; Department of Human Genetics, Leiden University Medical Center, Leiden, 2333 ZA, The Netherlands; Department of Human Genetics, Leiden University Medical Center, Leiden, 2333 ZA, The Netherlands; Department of Human Genetics, Leiden University Medical Center, Leiden, 2333 ZA, The Netherlands; Department of Human Genetics, Leiden University Medical Center, Leiden, 2333 ZA, The Netherlands; Department of Human Genetics, Leiden University Medical Center, Leiden, 2333 ZA, The Netherlands; Department of Human Genetics, Leiden University Medical Center, Leiden, 2333 ZA, The Netherlands; Institute of Biology Leiden, Leiden University, Leiden, 2333 BE, The Netherlands; Department of Human Genetics, Leiden University Medical Center, Leiden, 2333 ZA, The Netherlands

## Abstract

Polymerase theta-mediated end-joining (TMEJ) is a stand-alone mutagenic DNA double-strand break (DSB) repair pathway that becomes critical when high-fidelity repair is compromised. Although the enzymatic mechanism of TMEJ has been extensively studied in recent years, its regulation remains poorly understood. Here, we identify and characterize the RNA-binding protein Sam68 (also known as Khdrbs1) as a modulator of TMEJ in mammalian cells. We demonstrate that Sam68 is required for proper expression of *Polq*, the gene encoding polymerase theta (Polθ), the central factor in TMEJ; loss of Sam68 results in reduced TMEJ at CRISPR-induced DSBs. Mechanistically, Sam68 promotes correct splicing of *Polq* messenger RNA by suppressing the inclusion of a conserved poison exon (PE) that introduces a premature termination codon in the transcript. This function depends on the RNA-binding domain of Sam68. Genetic deletion of the PE restores *Polq* expression and rescues TMEJ activity in Sam68-depleted cells. Together, these findings establish alternative splicing as a direct mechanism controlling TMEJ capacity and reveal a conserved regulatory mechanism that tunes mutagenic DSB repair through modulation of Polθ abundance.

## Graphical Abstract

**Graphical Abstract:**
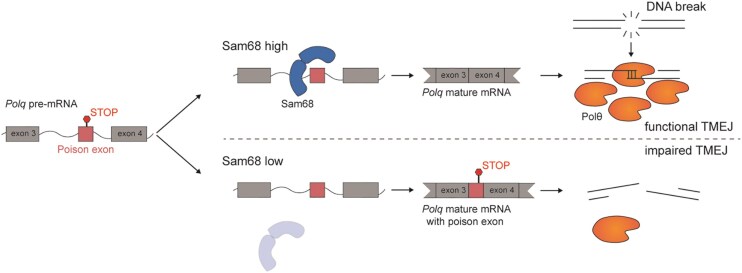

## Introduction

DNA double-strand breaks (DSBs) represent one of the most deleterious forms of genomic damage, and their repair is essential for maintaining genomic stability. Unrepaired or misrepaired DSBs can lead to cell death, mutations, and chromosomal rearrangements, contributing to cancer development and progression [1]. To avoid these detrimental effects, multiple repair pathways evolved to respond to DSBs, including polymerase theta-mediated end-joining (TMEJ). This pathway relies on the activity of DNA polymerase theta (Polθ), encoded by the *Polq* gene [2–4]. Polθ facilitates the annealing of short, complementary sequences (i.e. microhomology) present in 3′ protruding DSB ends that arise because of limited resection, followed by templated DNA synthesis to promote repair. As such, TMEJ is inherently error-prone and results in deletions characterized by microhomology at their junction; occasional primer-template switching by Polθ produces typical templated insertions [5, 6]. TMEJ functions as a critical DSB repair pathway in contexts where homologous recombination (HR) cannot be executed, most notably when the sister chromatid is unavailable or damaged. This situation arises when both sister chromatids sustain lesions, for example following ionizing radiation (IR) or upon persistent replication blocks, and is particularly relevant in HR-deficient cancers, where reliance on TMEJ underlies therapeutic vulnerabilities [7, 8]. Recent work further indicates that TMEJ plays an important role in the repair of DSBs that persist into mitosis, a cell-cycle phase in which HR is actively suppressed, and classical nonhomologous end joining (NHEJ) is constrained [9–14]. Consistent with these functions, TMEJ activity and Polθ expression are most prominent in fast-cycling cell types, including embryonic and germ cells as well as rapidly proliferating cancer cells, suggesting that TMEJ is especially important for maintaining genome integrity under conditions of high replicative and genotoxic stress [15, 16]. Under otherwise nonperturbed conditions, mutagenic TMEJ is indirectly suppressed by the more efficient and accurate NHEJ and HR pathways [4, 13]. However, whether there are also factors that directly suppress or stimulate TMEJ, e.g. by regulating DNA Polθ, remains largely unknown.

In a recent genetic “MUtational Scars of Induced DNA Cleavage” (MUSIC) screen for factors influencing DSB repair, Sam68 emerged as a candidate regulator of TMEJ outcomes [17]. Sam68 (Src-Associated In Mitosis 68 KDa Protein), also known as Khdrbs1 (KH RNA Binding Domain Containing, Signal Transduction Associated 1), is an RNA-binding protein previously implicated in alternative splicing, cell cycle regulation, and signal transduction [18]. In various cellular contexts, Sam68 has also been implicated in apoptosis and oncogenic transformation [18, 19]. Notably, high levels of Sam68 expression are found in multiple human cancers and inflammatory diseases, highlighting its potential role in disease progression and therapy [19, 20]. The impact of Sam68 on TMEJ mutational outcomes in the MUSIC screen prompted us to investigate its function in relation to this repair pathway.

## Materials and methods

### Cell culture

The 129/Ola-derived IB10 mouse embryonic stem (mES) cells were cultured in Buffalo rat liver-conditioned knockout Dulbecco’s modified Eagle’s medium (DMEM; Gibco). The knockout DMEM was supplemented with 100 U/ml penicillin, 100 μg/ml streptomycin, 2 mM GlutaMAX, 1 mM sodium pyruvate, 1 × nonessential amino acids, 100 μM β-mercaptoethanol (all from Gibco), 10% fetal calf serum (Capricorn Scientific), and leukemia inhibitory factor. The mES cell lines were maintained on 0.1% gelatin-coated plates. RPE1-hTERT (gift from Rob Wolthuis) and HEK293T cells were cultured in DMEM (Thermo Fisher Scientific) supplemented with penicillin/streptomycin (Sigma) and 10% fetal bovine serum (Bodinco BV) as previously described [7]. All cultures were incubated at 37°C with 5% CO_2_ and routinely tested for mycoplasma contamination.

### Generation of cell lines

*Ku80* ^−/−^ and *Polq*^−/−^ mES cells were previously described [21]. *Sam68*^−/−^ and *Polq*^+/−^ mES cell lines were created by transfecting wild-type HPRT-eGFP mES cells with plasmids that co-expressed Cas9-WT-2A-mCherry along with a *Sam68* or *Polq* targeting single guide RNA (sgRNA). To create *Sam68*^−/−^ mES cells, two independent sgRNAs were used to transfect two previously derived *HPRT-eGFP* wild-type clones. *Sam68-Polq* double-knockout mES cell lines were generated by targeting the *Sam68* gene in the already existing *HPRT-eGFP Polq*^−/−^ cell line. Two days post-transfection, mCherry-positive cells were sorted using a CytoFLEX SRT Cell Sorter (Beckman Coulter) and seeded at low density. The cells were maintained for 7 days to form colonies. Multiple clones from each transfection were picked and expanded. Clones with out-of-frame mutations were identified by Sanger sequencing of the targeted region, and protein expression levels of Sam68 were determined using western blot. CRISPR/Cas9-mediated deletion of the poison exon (PE) in *Polq* in mES cells was achieved by transfecting ribonucleoprotein (RNP) complexes. The Alt-R CRISPR RNAs (crRNAs) and trans-activating CRISPR RNAs (tracrRNAs) were resuspended in Nuclease-Free buffer, mixed in equimolar concentrations (10 µM), heated to 95°C for 5 min, and cooled to room temperature (RT). The crRNA:tracrRNA duplex was combined with Cas9 enzyme (1 µM) in Opti-MEM and incubated for 5 min at RT to form RNP complexes. For transfection, RNP complexes were mixed with RNAiMAX and Opti-MEM, incubated for 20 min, and then added to wild-type *HPRT-eGFP* mES cells. Cells were incubated for 48 h at 37°C, 5% CO_2_, with medium refreshed 24 h post-transfection. Afterwards, cells were seeded at low density. The cells were maintained for 7 days to form colonies. Multiple clones from each transfection were picked and expanded. Clones with bi-allelic PE loss were identified by Sanger sequencing. A detailed overview of the sgRNAs and polymerase chain reaction (PCR) primers used and the mutations introduced, can be found in Supplementary Table S1.

### Constructs

The plasmid pU6-(BbsI) CBh-Cas9-T2A-mCherry was provided by Ralf Kuehn (Addgene #64 324). pLenti-Cas9-2A-Blast was a gift from Jason Moffat (Addgene #73 310). Lentiviral packaging plasmids pMDLg/pRRE, pRSV-Rev, and pMD2.G were obtained from Didier Trono (Addgene #12 251, #12 253, #12 259). The pKLV2-U6gRNA5(BbsI)-PGKpuro2ABFP-W was a gift from Kosuke Yusa (Addgene #67 974). The pLentiGuide-eGFP backbone was kindly provided by Sylvie Noordermeer [22].

To generate constructs expressing the necessary sgRNAs, pairs of complementary oligonucleotides (Integrated DNA Technologies), containing the target sequences flanked by BbsI-compatible overhangs, were phosphorylated and annealed. These duplexes were then cloned into the BbsI-digested plasmids. Ligation was performed with T4 DNA ligase (Promega, Cat# M180B). All constructs were confirmed by Sanger sequencing. Detailed information about sgRNAs, plasmid destinations and sequencing primers is provided in Supplementary Table S2.

To generate piggyBac-based expression constructs for Sam68 variants, HA-tagged *Sam68* wild-type (pcDNA3 HA Sam68 WT, Addgene plasmid #17 690) and HA-tagged *Sam68ΔKH* (pcDNA3 HA Sam68 ΔKH, Addgene plasmid #17 688), were provided by David Shalloway. The coding sequences of these *Sam68* variants were amplified by PCR using primers incorporating SalI and AflII restriction sites (Supplementary Table S3). The obtained PCR products and the piggyBac expression vector (Addgene plasmid #153 521, a gift from Atsushi Miyawaki) were digested with SalI-HF and AflII (New England Biolabs) and subsequently ligated to generate the piggyBac-based expression plasmids.

### Lipofectamine transfection

As previously described [21], mES cells were trypsinized, counted, and resuspended in knockout DMEM (Gibco). Transfection was carried out in suspension using Lipofectamine 2000 (Invitrogen) at a 1:2.4 DNA to lipofectamine ratio. A total of 1.0 × 10^6^ cells were transfected with 2 µg of total DNA and incubated for 30 min at 37°C and 5% CO_2_ in round-bottom tubes. After incubation, the cells were plated onto gelatin-coated dishes with mES medium. The medium was changed 24 h after transfection.

### *HPRT-eGFP* mutation assay

As previously described [5, 7, 21], cells were transfected with a pU6-(BbsI) CBh-Cas9-T2A-mCherry plasmid, encoding the sgRNA that targets the *HPRT-eGFP* gene in exon 3. Two days following transfection, the cells were passaged, and the remainder of the cells was used to assess transfection efficiency. Transfection efficiency was determined by measuring the percentage of mCherry-positive cells using the NovoCyte flow cytometer (ACEA Biosciences, using NovoExpress software version 1.4.1). The mutation frequency (MF) was analyzed 5 days post-transfection on the NovoCyte by measuring the percentage of GFP-negative cells in the total population of cells. The relative MF was calculated by correcting for the transfection efficiency determined on day 2.

### Lentiviral production

Lentiviral production was performed in HEK293T cells using calcium phosphate transfection as previously described [17]. Cells were plated on T-175 flasks a day before transfection. The medium was changed for medium without penicillin/streptomycin 2 h before transfection. A mixture of plasmids was prepared, including 9 µg of the pMD2.G plasmid, 12.5 µg of the pMDLg/pRRE plasmid, 6.25 µg of the pRSV-Rev plasmid, and 32 µg of the transfer vector. These plasmids were diluted in 1125 µl of 0.1 × TE buffer [1 × TE: 10 mM Tris (pH 8.0) and 1 mM ethylenediaminetetraacetic acid (EDTA)]. To form precipitates, 125 µl of 2.5 M CaCl_2_ was added to the plasmid mixture, followed by a 5-min incubation at RT. Next, 1250 µl of 2 × HEPES-Buffered Saline (HBS) (pH 7.12) was added dropwise to the plasmid mixture while vortexing. The precipitate was then added to the cells. The medium was refreshed 16 h post-transfection. Thirty hours later, supernatant was collected and filtered through a sterile syringe filter with a Supor PES membrane (0.45 µm, 25 mm, PALL); aliquots were stored at −80°C. Viral titers were determined through serial dilution before use.

### Lentiviral transduction of mES and RPE1-hTERT cells

Stable expression of Cas9 was established in mES and RPE1-hTERT cell lines by transducing cells with the pLenti-Cas9-2A-Blast plasmid. Polybrene was added to the transduction medium at a final concentration of 8 µg/ml. Transduced cells were maintained under selection with blasticidin (5 µg/ml for mES cells; 10 µg/ml for RPE1-hTERT cells) before proceeding with experiments. To express the sgRNAs from the pKLV2-U6gRNA5(BbsI)-PGKpuro2ABFP-W backbone, cells were transduced with lentivirus at a multiplicity of infection (MOI) of 0.3 when sgRNAs were combined in a pooled experiment, while transductions where sgRNAs were individually delivered were performed at an MOI of > 1 (sgRNA sequences are provided in Supplementary Table S2). Polybrene was added to the transduction medium at a final concentration of 8 µg/ml. The day after transduction, cells were selected with 2 µg/ml puromycin for 3 days, with one passage during this period. After selection, the medium was refreshed, removing the puromycin. To generate repair outcome profiles within the pKLV2-U6gRNA5(BbsI)-PGKpuro2ABFP-W backbone, a second round of lentiviral transduction was performed using the pLentiGuide-eGFP construct expressing backbone-targeting sgRNA1 (pKLV2-U6gRNA5(BbsI)-PGKpuro2ABFP-W_targetsite_1). This transduction was performed at an MOI > 1. The medium was refreshed the next morning, and cells were harvested for DNA and/or RNA isolation ∼72 h post-transduction for the mES cells, and ∼120 h post-transduction for the RPE1-hTERT cells.

### Western blotting

Cells were lysed using 2% sodium dodecyl sulphate (SDS), 1% NP-40, 50 mM Tris (pH 7.5), and 150 mM NaCl in Milli-Q (MQ). Protein concentrations were determined using the Bicinchoninic Acid (BCA) Protein Assay Kit (Thermo Fisher Scientific) and adjusted accordingly. Protein samples were separated on 4%–12% Bis–Tris gradient gels (Novex) in MOPS SDS running buffer, with NuPAGE lithium dodecyl sulfate (LDS) sample buffer (all from Thermo Fisher Scientific), followed by transfer to Immobilon‐FL membranes (Merck Millipore). The following primary antibodies were used: anti-Tubulin (Sigma–Aldrich, T6199 clone DM1A, 1:5000), anti-SAM68 (Cell Signaling Technology, #12538, 1:1000) and anti-Ku80 (Santa Cruz, #sc-515736, 1:1000). Secondary antibodies, CF680-conjugated goat anti-rabbit IgG and CF770-conjugated goat anti-mouse IgG (Biotium, both at 1:10 000), were used, and protein expression was detected using the Odyssey CLx infrared imaging system (LI-COR Biosciences).

### Cell survival assay following IR

Clonogenic survival assays were performed in knockout cell lines by seeding trypsinized and counted cells at low density onto 60 mm culture dishes. Cells were irradiated using a YXlon X-ray generator (YXlon International) and cultured for 7 days to allow colony formation. Colonies were washed with 0.9% NaCl, stained with methylene blue, and manually counted. Surviving fractions were calculated relative to the cloning efficiency of untreated controls (0 Gy), which was normalized to 1.0 for each genotype.

For clonogenic survival assays following CRISPR-mediated gene depletion, the respective mES cell lines stably expressing Cas9 were transduced with lentiviral vectors expressing either a nontargeting sgRNA or sgRNAs targeting *Polq* or *Sam68* (sgRNA sequences are provided in Supplementary Table S2). Puromycin selection (2 µg/ml) was initiated 1 day after transduction and maintained for 4 days, with one passage during selection. Cells were seeded for clonogenic survival assays on day 5 after transduction. Irradiation, colony formation, and quantification were performed as described above.

### DNA isolation

Genomic DNA was extracted by lysing cell pellets in a buffer consisting of 10 mM Tris–HCl (pH 7.5), 10 mM EDTA, 200 mM NaCl, 1% SDS, and 0.4 mg/ml Proteinase K. The lysate was incubated at 55 °C for 3–16 h, followed by neutralization with saturated NaCl. The solution was then centrifuged at 20 000 × *g* for 10 min. DNA was precipitated by adding an equal volume of isopropanol to the supernatant and then subjected to centrifugation. The resulting DNA pellet was washed with 70% ethanol and resuspended in MQ.

### Targeted sequencing of repair outcomes

For targeted sequencing of the pKLV2-U6gRNA5(BbsI)-PGKpuro2ABFP-W backbone, DNA samples were prepared as described in [17]. In brief, NEBNext Ultra II Q5 Master Mix (NEB) was used in a 50 µl reaction mixture containing 500 ng genomic DNA and 0.2 µM forward and reverse primers. The PCR program included an initial denaturation at 98 °C for 3 min, followed by 18 cycles of 98 °C for 10 s, 62 °C for 30 s, and 72 °C for 45 s. The reaction concluded with a final extension at 72 °C for 5 min. The first PCR product was purified using AMPure XP magnetic beads (Beckman Coulter) at a 0.8× ratio and eluted in MQ.

The second round of PCR was aimed to amplify the desired fragment, incorporate the adaptors, and add the index sequences for Illumina sequencing. PCR2 was performed using NEBNext Ultra II Q5 Master Mix (NEB), 5 µl of purified PCR1 as a template and 0.2–0.4 µM of each primer was used. The cycling conditions were as follows: initial denaturation at 98 °C for 2 min, followed by 10 cycles of 98 °C for 10 s, 65 °C for 75 s, and a final extension at 65 °C for 5 min. The PCR2 product was purified using AMPure XP beads at a 0.8× ratio and eluted in MQ.

For targeted sequencing of the *HPRT-eGFP* locus, samples were processed as described in [21]. In brief, primers specific to the target regions were designed to amplify a 150–200 bp product. Amplification was performed using NEBNext Ultra II Q5 Master Mix (NEB) with the following cycling conditions: initial denaturation at 95 °C for 10 min, followed by 25 cycles of 95 °C for 10 s, 60 °C for 30 s, and 72 °C for 30 s, with a final extension at 72 °C for 5 min. PCR products were purified using AMPure XP magnetic beads (Beckman Coulter) at a 1.2× ratio and eluted in 20 µl MQ.

To add the indexes for Illumina sequencing, a second PCR was performed using 5 µl of the purified PCR1 product, 0.3 µM of the p5 and p7 index primers, and Phusion High-Fidelity DNA Polymerase (Thermo Fisher Scientific). The conditions included an initial denaturation at 95 °C for 10 mins, followed by five cycles of 95 °C for 10 s, 58 °C for 30 s, and 72 °C for 30 s, with a final extension at 72 °C for 5 min. The PCR products were purified using AMPure XP beads at a 0.8× ratio and eluted in 20 µl MQ.

Yield was measured using the Qubit double-stranded DNA High Sensitivity Assay (Thermo Fisher Scientific), and sample quality was checked on a Bioanalyzer (Agilent). Samples were pooled in equimolar amounts, and the final library was sequenced by 150-bp paired-end sequencing on a NovaSeq 6000 or NovaSeq X (Illumina). Primer and adaptor sequences are provided in Supplementary Table S2.

### RNA isolation, complementary DNA synthesis, and targeted sequencing of complementary DNA

Total RNA was isolated from cells using the RNeasy mini kit (Qiagen). Then, 1 μg of total RNA was reverse transcribed into complementary DNA (cDNA) using the PrimeScript RT reagent kit (Takara Bio). cDNA was amplified by PCR to visualize the region around the *Polq* PE on a 2% agarose gel. To quantify PE inclusion using next-generation sequencing, we followed the targeted sequencing protocol that was used for sequencing of the *HPRT-eGFP* locus (see above), with one adjustment: the first amplification was carried out using Gotaq polymerase (Promega). An overview of the primers used for PCR and Next-Generation Sequencing (NGS) can be found in Supplementary Table S3.

### RNA sequencing

For RNA sequencing, total RNA was extracted from mES cells using the NucleoSpin RNA Mini Kit (Macherey-Nagel), following the manufacturer’s protocol. RNA concentration and purity were assessed using a NanoDrop spectrophotometer, and RNA integrity was evaluated using the Agilent 2100 Bioanalyzer with RNA 6000 Nano chips (Agilent Technologies) to determine RNA Integrity Numbers (RIN). Only samples with RIN ≥ 9 were selected for downstream processing. To enable normalization and monitoring of technical variation, the ERCC RNA Spike-In Mix (Thermo Fisher Scientific) was added to each sample prior to library preparation.

RNA sequencing libraries were prepared by Macrogen Inc. (Seoul, South Korea) using the TruSeq Stranded Total RNA Library Prep Kit with Ribo-Zero Human/Mouse/Rat (Illumina). Libraries were sequenced on an Illumina platform (NovaSeq X Plus) with 150 bp paired-end read configuration.

### Total RNA sequencing analyses

Sequences were trimmed using TrimGalore-0.6.5 with quality setting 30 and Cutadapt version 2.9 [23]. Trimmed reads were first mapped to GRCm39_MusMusculus using STAR (v2.7.7a) [24] with settings -sjdbGTFfile Mus_musculus.GRCm39.114.gtf and -outSAMmultNmax 1. For optimal quantification of alternative splicing events between samples, trimmed reads were re-mapped to GRCm39_MusMusculus using STAR (v2.7.7a), this time using settings -outSJfilterReads Unique, -outSAMstrandField intronMotif, -outFilterMultimapNmax 1, and providing a list of all individual SJ.out.tab files obtained from the first STAR runs in -sjdbFileChrStartEnd. Track figures around *Polq* exon 3 and 4 were generated in IGV (v2.19.6) [25].

For subsequent alternative splicing analyses, reads mapping to alternative chromosome patches were removed using Samtools (version 1.11) [26]. Splicing analyses were performed using Junction Usage Model (JUM; v3.0.0) [27], testing three wild-type versus three *Sam68*^−/−^ samples. Included were splice junctions that received more than 10 unique mapped reads in at least 2 of the 3 samples per condition (5 for intron retention events). All alternative splicing events were obtained using *P*-value setting 1. Read length was defined as 150bp. Alternative splicing events were annotated in JUM-C using a refFlat_GRCm39-file downloaded from the UCSC Table Browser (https://genome.ucsc.edu/cgi-bin/hgTables). Downstream data processing was performed in Excel. Delta percent spliced in (ΔPSI) values represent cassette exon inclusion frequency of wild-type minus *Sam68*^−/−^; negative ΔPSI means more inclusion in *Sam68*^−/−^ compared to wild-type; positive ΔPSI means less inclusion in *Sam68*^−/−^ compared to wild-type.

RNA sequencing data from [28] were obtained from GEO with accession number GSE153800 (GSM4654311–GSM4654319; *Sam68*^−/−^, GSM4654329–GSM4654337; wild type) and were analyzed in the same way. JUM analysis data can be found in Supplemental Tables S5–S7.

### Next-generation sequencing analysis

Next-generation sequencing data were processed using a custom JAVA program (SIQ), with the resulting output analyzed and visualized using the SIQplotteR web tool [29]. In pooled experiments, the design allowed the repair outcome to be identified from one end of the sequencing read, while the sgRNA sequence could be determined from the other end (as previously described in [17]). For these samples, paired-end reads were first demultiplexed based on an exact match to the sgRNA. The processed files were then analyzed with SIQ. To quantify the TMEJ/NHEJ ratio for the HPRT exon 3 target site the fraction of two TMEJ-driven events (a 14 bp deletion with 6 bp microhomology and a 5 bp deletion with 3 bp microhomology) is divided by the fraction of an NHEJ-driven 1 bp insertion. To quantify the TMEJ/NHEJ ratio for the sgTargeting-1 target site the fraction of two TMEJ-driven events (a 11 bp deletion with 4 bp microhomology usage and a 6 bp deletion with 3 bp microhomology usage) is divided by an NHEJ-driven 1 bp insertion.

### Statistical analysis

Statistical analyses were performed in GraphPad Prism. Normality of residuals was assessed by visual inspection. Ratio-based measures were log-transformed prior to analysis to improve normality of residuals. For experiments comparing more than two groups, one-way Analysis of Variance (ANOVA) was used, followed by Dunnett’s multiple comparisons test when comparing multiple groups to a single control, or with Šidák’s correction when specific pairwise comparisons between genotypes were made. For direct comparisons between two groups only, a two-tailed unpaired *t*-test was applied. A *P*-value below 0.05 was considered statistically significant.

## Results

### MUSIC identifies Sam68 as a stimulator of TMEJ-driven repair outcomes

**Figure 1.**
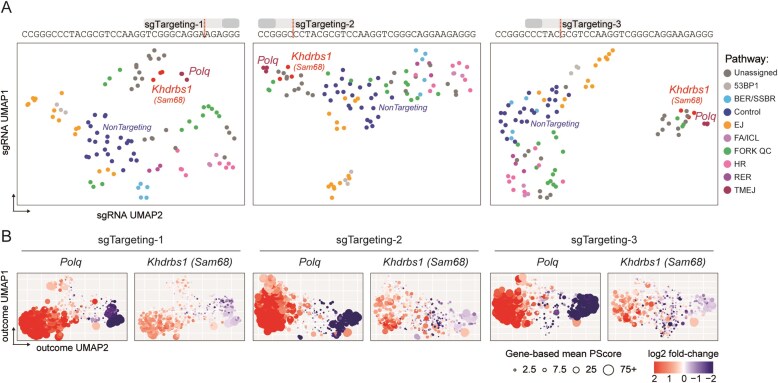
*Khdrbs1* (*Sam68*) sgRNAs cluster with *Polq* sgRNAs and reduce TMEJ-outcomes in MUSIC data. (**A**) Using custom SIQ software [29], the deconvolution of a subset of the published MUSIC dataset [17] produces a UMAP projection of the top outlier sgRNAs for deviation in mutational signatures at three different CRISPR/Cas9 target sites (sgTargeting-1, sgTargeting-2, and sgTargeting-3). Each dot represents an individual sgRNA, positioned based on the similarity of its repair outcome distribution to that of other outliers and nontargeting sgRNAs. Colors represent the DNA repair pathway associated with each sgRNA. sgRNAs targeting *Polq* and *Khdrbs1* (*Sam68*) are highlighted (see Supplemental Fig. S1A for a fully annotated UMAP representation). (**B**) UMAP projection of mutational outcomes for the three different CRISPR/Cas9 target sites. The log_2_ fold-change in repair outcomes for *Polq* and *Khdrbs1* (*Sam68*) sgRNAs (average of 3 sgRNAs per gene) relative to nontargeting sgRNAs is shown; dots are sized based on PScore. Absent repair outcomes are represented as small white dots.

Recently, we employed a CRISPR/Cas9-based high-throughput knockout approach to obtain a comprehensive catalogue of MUSIC, mapping the entire set of genes involved in DSB repair [17]. We used mES cells to at high resolution determine potential changes in mutational signatures at CRISPR/Cas9-induced DSBs upon the genetic perturbation of ∼800 genes using a candidate gene sgRNA library. After generating polyclonal knockout populations, the library backbone was separately targeted at three unique sgRNA target sites (sgTargeting-1, sgTargeting-2, and sgTargeting-3) in close proximity of the gene-specific sgRNA. This experimental design allows the coupling of a repair outcome (at the targeted site) to the genetic background of the cell in which that repair took place (using the sgRNA sequence as a barcode). To visualize and summarize this data, we applied Uniform Manifold Approximation and Projection (UMAP). In this UMAP, sgRNAs with similar mutagenic outcome redistribution patterns group together. Notably, we found that sgRNAs targeting *Polq* and *Khdrbs1* (from now on referred to as *Sam68*) clustered closely together in the UMAP of all three target-sites, suggesting that DSB repair upon perturbation of *Polq* or *Sam68* is affected in a similar manner (Fig. 1A and Supplementary Fig. S1A, for clarity only a subset of genes are depicted in Fig. 1A). We next analyzed the mutagenic outcomes at these three Cas9 target sites in *Polq*- or *Sam68*-depleted cells; complementary UMAPs in which repair outcomes are clustered based on shared genetic dependencies in the MUSIC screen reveal that *Sam68*-depleted cells strongly resemble *Polq*-depleted cells (Fig. 1B), albeit the deviation from nontargeting sgRNAs less pronounced. The changes include a decrease in microhomology-driven (*Polq*-dependent) deletions and an increase in NHEJ-mediated (*Lig4*-dependent) mutations (Fig. 1B and Supplementary Fig. S1B). We conclude that Sam68 stimulates mutagenic repair of DSBs by TMEJ.

### *Sam68* knockout cells show reduced TMEJ

**Figure 2.**
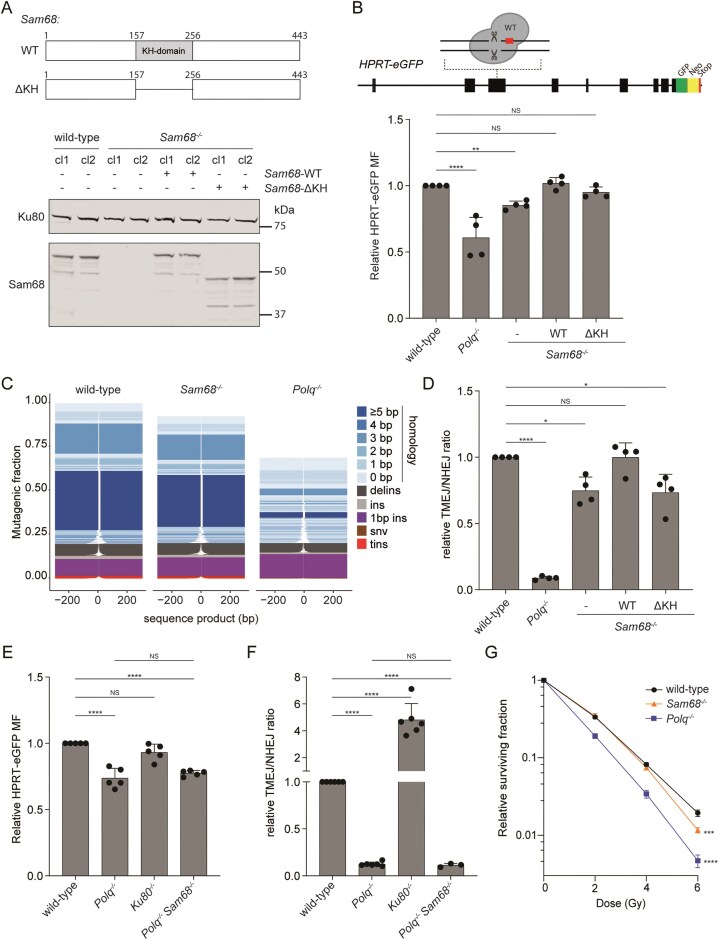
*Sam68* knockout mES cells display reduced TMEJ activity. (**A**) Graphic illustration of cDNA constructs expressing mouse Sam68WT and Sam68ΔKH (top), followed by western blot analysis showing the loss of Sam68 protein expression in two independent *Sam68* knockout clones and the complementation of these knockout clones using cDNA constructs expressing mouse Sam68WT and Sam68ΔKH (bottom). Ku80 serves as a loading control. cl1, clone 1; cl2, clone 2. (**B**) Top: Schematic representation of the *HPRT-eGFP* gene with the *HPRT* exon 3 CRISPR/Cas9 target site used in this study. Bottom: Relative *HPRT-eGFP* MF in the indicated mES cell lines transfected with Cas9-WT and the sgRNA targeting *HPRT* exon 3. Data are shown as mean ± standard deviation (SD; *n* = 4), with values expressed as a fraction of the MF observed in wild-type cells (set to 1). Statistical significance was determined using one-way ANOVA followed by Dunnett’s multiple comparisons test; ns, not significant, **P ≤.01, ****P ≤.0001. (**C**) Mutational signatures at Cas9-induced DSBs for the indicated mES cell lines transfected with Cas9-WT and the sgRNA targeting *HPRT* exon 3. Cells were collected for DNA extraction and used for targeted sequencing around the break site. Data are represented as tornado plots where the height of each bar reflects the contribution of each outcome to the total amount of reads within the dataset. Data are plotted relative to the Cas9-WT break site (0) and sorted by mutational type. Mutation types are color-coded. The degree of blue coloring reflects the extent of microhomology present at the deletion junction. Data are from four independent experiments (*n* = 4). delins, deletion insertion; snv, single nucleotide variant; ins, insertion; tins, templated insertions; bp, base pair. (**D**) Quantification of TMEJ/NHEJ mutagenic events obtained by targeted sequencing (see the ‘Materials and methods’ section for details). The ratio of TMEJ/NHEJ mutagenic events in wild-type cells is set to 1. Data are shown as mean ± SD (*n* = 4). Ratio-based measures were log-transformed prior to analysis. Statistical significance was determined using one-way ANOVA followed by Dunnett’s multiple comparisons test; ns, not significant, *P ≤.05, ****P ≤.0001. (**E**) Similar to panel (B), using indicated genotypes. Data are shown as mean ± SD (*n* = 5). Statistical significance was determined using one-way ANOVA followed by Šidák’s multiple comparisons test; ns, not significant and ****P ≤.0001. (**F**) Similar to panel (D), using indicated genotypes. Data are shown as mean ± SD (*n* = 3 or 6). Ratio-based measures were log-transformed prior to analysis. Statistical significance was determined using one-way ANOVA followed by Šidák’s multiple comparisons test; ns, not significant and ****P ≤.0001. (**G**) Clonogenic survival of the indicated mES cell lines following exposure to varying doses of IR. Data are presented as mean ± standard error of the mean (*n* = 12, with three independent experiments using two clones per genotype and two technical replicates per clone). Statistical significance compared to wild-type was determined using one-way ANOVA followed by Dunnett’s multiple comparisons test; ***P ≤.001 and ****P ≤.0001.

To substantiate the outcomes obtained using MUSIC, we generated monoclonal *Sam68* mES knockout cells. Knockout was confirmed by western blot and two clones were selected for further validation (Supplementary Fig. S2A). As additional controls, we complemented the *Sam68* knockout cells with full-length mouse *Sam68* (Sam68WT) as well as a mutant construct lacking the K-homology domain (Sam68ΔKH) (Fig. 2A), which is essential for binding to RNA [30]. We subsequently monitored mutagenic DNA repair at CRISPR/Cas9-induced breaks in these cells. To this end, we integrated the eGFP-coding sequence into the *HPRT* locus, creating a functional eGFP-tagged *HPRT* gene [5], which allows for establishing the mutation frequency (MF) at any DSB site within the *HPRT-eGFP* open reading frame by quantifying the percentage of GFP-negative cells in the population after targeting this region using CRISPR/Cas9 technology (Fig. 2B). Upon the introduction of a DSB with Cas9-WT in *HPRT* exon 3, *Sam68* knockout clones showed a reduction in MF compared to wild-type cells, albeit less pronounced compared to *Polq* knockout clones (Fig. 2B). We next performed targeted sequencing of bulk-transfected cells to compare the DSB repair profiles of Sam68- and Polθ-deficient cells with those of wild-type cells. As previously established, knocking-out *Polq* leads to a reduction in deletions characterized by junctional microhomology, whilst increasing 1-base pair insertions that are associated with NHEJ [5] (Fig. 2C and D, and Supplementary Fig. S2B). In *Sam68* knockout cells, we similarly observe a reduction in TMEJ outcomes and an increase in NHEJ-associated mutagenesis, indicating that TMEJ is impaired in the absence of Sam68 (Fig. 2C and D, and Supplementary Fig. S2B). The reduction in TMEJ outcomes in Sam68-deficient cells can be rescued by reintroduction of full-length *Sam68* mouse cDNA (Sam68WT) but not by Sam68ΔKH (Fig. 2D and Supplementary Fig. S2B). Notably, the expression of these proteins in wild-type cells had no effect on the MF or mutational signatures at Cas9-induced breaks (Supplementary Fig. S2C–E). To determine whether *Sam68* and *Polq* are epistatic, we generated double knockout cells lacking both genes. By comparing the MF and mutational signatures at a CRISPR/Cas9-induced DSB, we found that *Polq*^−/−^  *Sam68*^−/−^ cells phenocopied the *Polq*^−/−^ cells, demonstrating that loss of Sam68 specifically reduces TMEJ-dependent mutagenic outcomes (Fig. 2E and F, and Supplementary Fig. S2F). Furthermore, we found Sam68-deficient cells to be more sensitive to high-doses of IR than wild-type cells, suggesting that Sam68, like Polθ, also stimulates the repair of IR-induced DNA damage (Fig. 2G). Of note, we cannot exclude that the increased IR sensitivity of *Sam68* knockout cells extends beyond its effect on TMEJ as Sam68 has also been reported to modulate other DNA repair factors [31, 32]. We conclude that Sam68 is important for TMEJ proficiency, via its RNA-binding capacity.

### Sam68 prevents the inclusion of a PE in the *Polq* transcript

**Figure 3.**
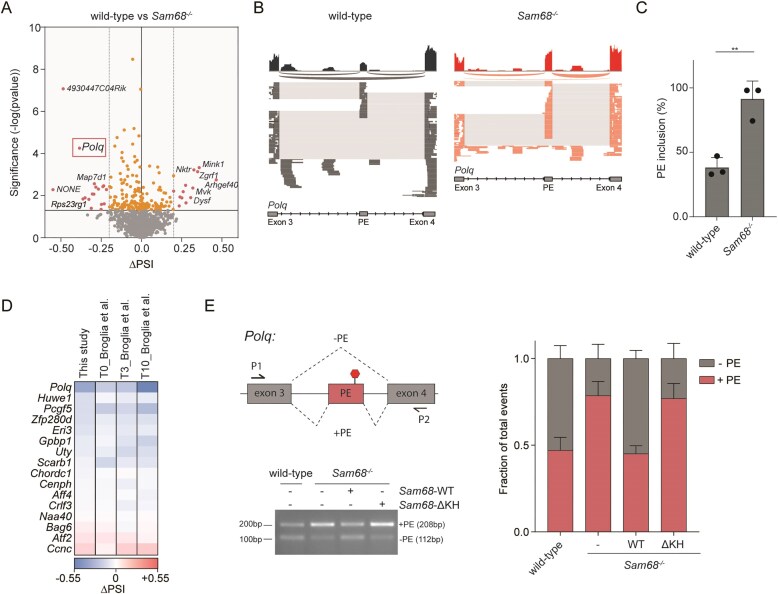
Sam68 suppresses PE inclusion in the *Polq* transcript. (**A**) Volcano plot showing differentially cassette exon usage in mES wild-type cells versus mES *Sam68*^−/-^ cells based on RNA-seq data. Each dot represents a cassette exon event, with the *x*-axis showing ΔPSI (the difference in proportion of cassette exon usage between the two groups) and the *y*-axis representing significance [−log₁₀(*P*-value)]. Positive ΔPSI values indicate more inclusion of the cassette exon in the wild-type cells, while negative values indicate more inclusion in the *Sam68* knockout cells. Events that are statistically significant with a modest effect size (−0.2 ≤ ΔPSI ≤ 0.2) are shown in orange, whereas statistically significant events with a strong (ΔPSI < −0.2 or ΔPSI > 0.2) effect are shown in red. Nonsignificant events are shown in grey. Gene associations were determined by JUM-analyses [27]. (**B**) Sashimi plots of three replicates combined showing RNA-seq read coverage and exon–exon junction usage for the PE event in the *Polq* transcript for wild-type and *Sam68* knockout cells. (**C**) PE inclusion (%) in the *Polq* transcript in wild-type and *Sam68* knockout cells. Data represent the percentage of PE inclusion, calculated from three biological replicates of RNA-seq data. Data are shown as the mean ± SD (*n* = 3); **P ≤.01. (**D**) Heatmap of ΔPSI values for cassette exon events across our dataset and the published embryoid body differentiation dataset (days 0, 3, and 10) [28]. The 16 cassette exon events shown are found in all four datasets. Color indicates the magnitude and direction of ΔPSI, with positive (red) values reflecting increased PE inclusion in wild-type cells and negative values (blue) reflecting increased PE inclusion in *Sam68* knockout cells. (**E**) Schematic representation of the PE and primers used for RT-PCR in the mouse *Polq* gene. The ratio of PE inclusion/exclusion in *Polq* was determined in the indicated mES cell lines using RT-PCR followed by agarose analysis, and short-read targeted sequencing of the cDNA. Data are shown as mean ± SD (*n* = 4).

Since Sam68 is a known splicing factor, we hypothesized that it may stimulate TMEJ by regulating the processing of *Polq* transcripts. To study this, we performed total RNA sequencing (RNA-seq) on wild-type and *Sam68* knockout mES cells and carried out a JUM splicing analysis. As previously published [28, 33], Sam68 depletion results in abundant differentially regulated splicing events (Supplementary Fig. S3A). Upon analysis of the different classes of alternative splicing affected by Sam68 loss, we detected *Polq* as the second most significantly affected gene in the ‘cassette exon’ group (after *930447C04Rik*, representing meiotic protein SIX6OS1), with a ΔPSI value of −0.385 for wild-type versus *Sam68*^−/−^ cells (Fig. 3A). JUM identifies cassette exons by analyzing splice junction connectivity to detect exon inclusion and skipping junctions. The cassette exon event detected in *Polq* corresponds to a poison exon (PE) located between exons 3 and 4, whose inclusion introduces a premature stop codon (Fig. 3B and Supplementary Fig. S3B). PE inclusion likely promotes nonsense-mediated RNA decay [34]; potential residual expression from this transcript results in a truncated protein containing only ∼6% of the coding sequence and lacking any functional or binding domains of Polθ (Supplementary Fig. S3C). In wild-type cells, Sam68 suppresses the inclusion of this PE in *Polq* transcripts, as knocking-out *Sam68* results in a strong increase in PE inclusion: 91.5% PE-inclusion in *Sam68*^−/−^ cells versus 38.3% in wild-type cells (Fig. 3C). To substantiate our finding, we compared our data to a previously deposited RNA-seq dataset from *Sam68* knockout mES cells [28]. In this study, wild-type and *Sam68*^−/−^ mES cells were allowed to differentiate into tissue-like spheroids during which RNA was isolated at three different time-points (days 0, 3, and 10). Re-analysis of this dataset revealed that the inclusion of the *Polq*-PE was the top hit regulated by Sam68, with the highest ΔPSI value at day 10 (Fig. 3D and Supplementary Fig. S3D). Next, we aimed to confirm increased PE inclusion in *Polq* transcripts in *Sam68* knockout cells and study complementation. To that end we performed reverse transcriptase-polymerase chain reaction (RT-PCR) followed by agarose gel analysis and next-generation sequencing on cDNA from this region in wild-type, *Sam68* knockout cells, and *Sam68* knockout cells complemented with either Sam68WT or Sam68ΔKH (Fig. 3E). Confirming the RNA-seq data, we found already substantial levels of PE inclusion in wild-type mES cells (50% of transcripts). Loss of Sam68, however, further increased PE inclusion in *Polq* transcripts (up to ∼75%), which was rescued by re-expression of Sam68WT but not Sam68ΔKH in *Sam68^−/−^* cells (Fig. 3E). Interestingly, ectopic expression of Sam68WT, but not Sam68ΔKH, in wild-type cells led to a further reduction in PE-inclusion (Supplementary Fig. S3E).

### The *Polq* PE and its regulation by Sam68 are evolutionary conserved

**Figure 4.**
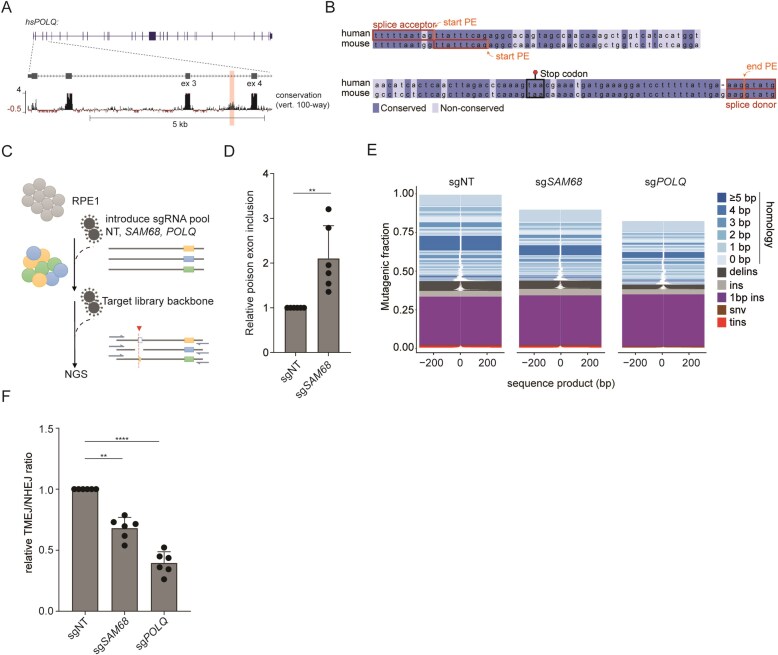
Sam68-mediated regulation of *POLQ* PE-inclusion is evolutionary conserved. (**A**) Conservation analysis of the PE in the human *POLQ* gene across vertebrates based on phyloP basewise conservation scores derived from the Multiz 100-way vertebrate alignment using the UCSC Genome Browser. The PE region displayed elevated positive phyloP scores relative to the surrounding intronic sequences, indicating strong evolutionary constraint across vertebrate species. (**B**) Pairwise sequence alignment of the *POLQ* PE region (based on our cDNA sequence data) between human and mouse generated using Jalview software [36]. Conserved nucleotides are indicated in dark blue, while nonconserved nucleotides are shown in light blue. The splice acceptor and splice donor sites are highlighted in red. The start and end positions of the PEs are marked in orange. (**C**) Schematic overview of the experimental workflow. Wild-type RPE1 cells expressing Cas9 were transduced with lentiviral vectors carrying sgRNAs targeting *SAM68* and *POLQ* or nontargeting control sgRNAs. Following CRISPR/Cas9-mediated DNA cleavage at the library backbone target site, mutational outcomes were assessed by targeted sequencing. (**D**) Quantification of PE inclusion in *POLQ* cDNA using targeted sequencing. PE inclusion in wild-type cells is set to 1.0. Data are shown as mean ± SD (*n* = 6). Statistical significance was determined using a two-tailed unpaired *t*-test; **P  ≤.01. (**E**) Mutational signatures at Cas9-induced DSBs in RPE1 cells depleted for *POLQ* or *SAM68* or nontargeting control (*n* = 6). Cells were collected for DNA extraction and used for short-read targeted sequencing around the break site in the library backbone. Data are represented as tornado plots where the height of each bar reflects the contribution of each outcome to the total amount of reads within the dataset. Data are plotted relative to the Cas9-WT break site (0) and sorted by mutational type. Mutation types are color-coded. The degree of blue coloring reflects the extent of microhomology present at the deletion junction. (**F**) Quantification of TMEJ/NHEJ mutagenic events obtained by targeted sequencing (see the ‘Materials and methods’ section for details). The ratio of TMEJ/NHEJ mutagenic events in wild-type cells is set to 1. Data are shown as mean ± SD (*n* = 6). Ratio-based measures were log-transformed prior to analysis. Statistical significance was determined using one-way ANOVA followed by Dunnett’s multiple comparisons test; **P ≤.01 and ****P ≤.0001.

Having established that Sam68 suppresses PE inclusion in *Polq* transcripts in mES cells, we next wondered whether this regulation is evolutionary conserved. To assess this question, we examined phyloP basewise conservation scores derived from the Multiz 100-way vertebrate alignment using the UCSC Genome Browser [35]. The *POLQ* region containing the PE displayed elevated positive phyloP scores relative to the surrounding intronic sequences, indicating strong evolutionary constraint across vertebrate species (Fig. 4A). Notably, conservation extended across the exon body and splice junctions, consistent with functional importance of this regulatory element (Fig. 4A and B). In agreement, the *POLQ*-PE was previously identified in a study using deep sequencing of pre-translational mRNA-protein particles (mRNPs) in human cells, which also suggests that its inclusion is coupled to nonsense-mediated decay-associated alternative splicing outcomes [34].

To test whether Sam68 also suppresses PE-inclusion (and thus stimulates TMEJ) in human cells, we lentivirally delivered sgRNAs targeting *SAM68, POLQ* or nontargeting sgRNAs to wild-type RPE1-hTERT cells stably expressing Cas9 (Fig. 4C). Efficient depletion of Sam68 in polyclonal cell populations was confirmed by western blot (Supplementary Fig. S4A). Subsequently, and following the MUSIC approach [17], the lentiviral backbone was targeted using CRISPR/Cas9 to introduce a single DSB in the vicinity of the sgRNA, which served as a barcode. Six days after targeting cells were harvested for RNA isolation to assess PE-inclusion rates, and for DNA isolation to analyze the mutational profile of DSB repair using NGS. We found that Sam68 depletion in RPE1 cells caused a two-fold increase in PE inclusion in human *POLQ* transcripts relative to nontargeting controls, demonstrating that the regulation of *POLQ* splicing via Sam68 is conserved in human cells (Fig. 4D). The depletion of Sam68 in RPE1 cells resulted in a reduction in both the MF as well as TMEJ-associated mutational outcomes (Fig. 4E and F), establishing that Sam68 also stimulates TMEJ in human cells.

### Genomic deletion of the *Polq* PE rescues TMEJ activity in Sam68-deficient cells

**Figure 5.**
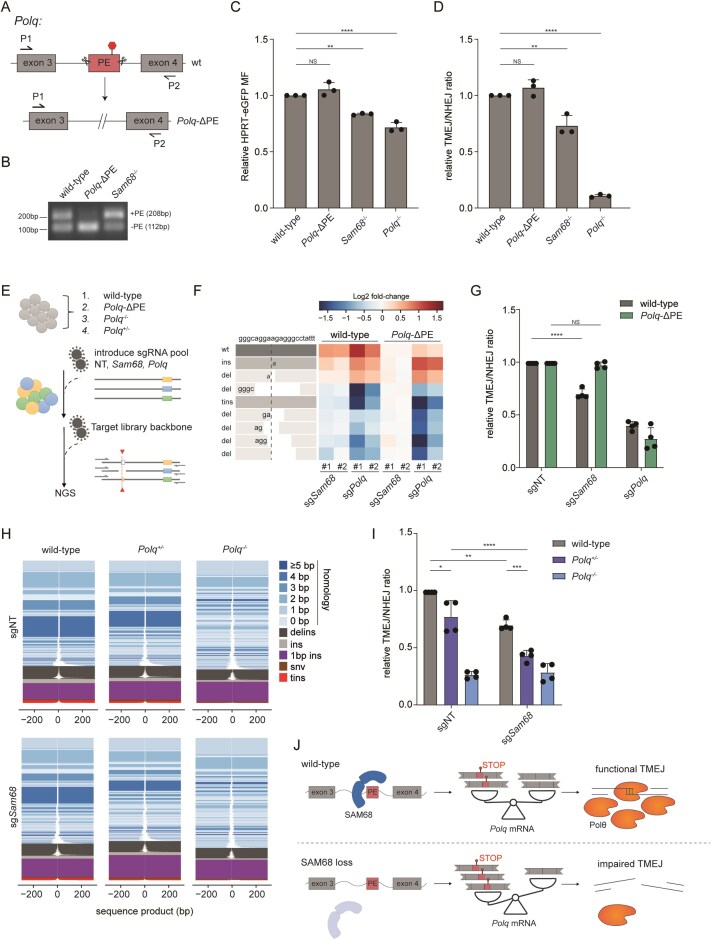
Genomic deletion of *Polq*-PE rescues Sam68-phenotype. (**A**) Schematic illustration of the generation of a mES cell line with the PE of *Polq* deleted (*Polq*-ΔPE). PE deletion was achieved through the co-delivery of Cas9 protein and two sgRNAs. (**B**) The deletion of the PE in *Polq* was confirmed by RT-PCR analysis, followed by agarose gel electrophoresis. (**C**) Relative *HPRT-eGFP* MF in the indicated mES cell lines transfected with Cas9-WT and the sgRNA targeting *HPRT* exon 3. Data are shown as mean ± SD (*n* = 3), with values expressed as a fraction of the MF observed in wild-type cells (set to 1). Statistical significance was determined using one-way ANOVA followed by Dunnett’s multiple comparisons test; ns, not significant, **P ≤.01, ****P ≤.0001. (**D**) Quantification of TMEJ/NHEJ mutagenic events obtained by targeted sequencing. The ratio of TMEJ/NHEJ mutagenic events in wild-type cells is set to 1. Data are shown as mean ± SD (*n* = 3). Ratio-based measures were log-transformed prior to analysis. Statistical significance was determined using one-way ANOVA followed by Dunnett’s multiple comparisons test; ns, not significant, **P ≤.01, ****P ≤.0001. (**E**) Schematic overview of the experimental workflow. The indicated mES cell lines were engineered to express Cas9 and were subsequently transduced with lentiviral vectors carrying sgRNAs targeting *Sam68* and *Polq* or nontargeting control sgRNAs. Following CRISPR/Cas9-mediated DNA cleavage at the target site in the library backbone, mutational outcomes were assessed by targeted sequencing. (**F**) Mutational outcomes at the Cas9-induced DSB for the indicated Cas9-expressing mES cell lines transduced with the sgRNA targeting the library backbone. Cells were collected for DNA extraction and used for targeted sequencing around the break site. A heatmap of the top nine altered mutational outcomes relative to the nontargeting control is shown. Rows represent mutational outcomes, while columns represent experimental conditions. The color scale indicates the log_2_ fold-change relative to the nontargeting control. (**G**) Quantification of TMEJ/NHEJ mutagenic events obtained by short-read targeted sequencing. The ratio of TMEJ/NHEJ mutagenic events in wild-type cells is set to 1. Data are shown as mean ± SD (*n* = 4). Ratio-based measures were log-transformed prior to analysis. Statistical significance was determined using one-way ANOVA followed by Šidák’s multiple comparisons test; ns, not significant and ****P ≤.0001. **(H)** Mutational signatures at the Cas9-induced DSB for the indicated Cas9-expressing mES cell lines transduced with the sgRNA targeting the library backbone. Cells were collected for DNA extraction and used for short-read targeted sequencing around the break site. Data are represented as tornado plots where the height of each bar reflects the contribution of each outcome to the total amount of reads within the dataset. Data are plotted relative to the Cas9-WT break site (0) and sorted by mutational type. Mutation types are color-coded. The degree of blue coloring reflects the extent of microhomology present at the deletion junction (*n* = 4). (**I**) Similar to panel (G), using indicated genotypes and conditions. Data are shown as mean ± SD (*n* = 4). Ratio-based measures were log-transformed prior to analysis. Statistical significance was determined using one-way ANOVA followed by Šidák’s multiple comparisons test; *P ≤.05, **P ≤.01, ***P ≤.001, ****P ≤.0001. (**J**) Model for the role of Sam68 in regulating *Polq* expression and TMEJ activity. Sam68 modulates the splicing of *Polq* pre-mRNA by repressing the inclusion of a PE, which would otherwise lead to a premature termination codon. Loss of Sam68 disrupts this regulation, resulting in reduced functional Polθ expression and diminished TMEJ activity.

The data thus far suggests that Sam68 stimulates TMEJ by suppressing PE inclusion in *Polq* messenger RNA (mRNA). To provide experimental evidence for this hypothesis and exclude the possibility that Sam68 regulates TMEJ in other ways, we removed the PE sequence from the *Polq* gene in mES cells. Using CRISPR/Cas9 and two sgRNAs flanking the PE-region (Fig. 5A) we deleted the PE in both alleles generating *Polq*-ΔPE cells. Successful removal was confirmed on the DNA level by Sanger sequencing and on the RNA level by RT-PCR (Supplementary Fig. S5A and B). To assure that removal of the PE does not negatively interfere with TMEJ activity, we determined the MF and mutational signatures at a DSB introduced in the *HPRT-eGFP* gene. Instead, we observed a slight (although not significant) increase in MF as compared to wild-type cells (Fig. 5C). Next-generation sequencing around the break-site also revealed a slight increase in TMEJ-mediated mutations compared to wild-type cells, potentially reflecting higher levels of Polθ expression (Fig. 5D and Supplementary Fig. S5B).

Having established that endogenous removal of the PE sequence did not impair TMEJ, we next tested the prediction that TMEJ in these cells is insensitive to Sam68 loss, as all *Polq* transcripts now lack the substrate onto which Sam68 acts. Cas9-expressing wild-type and *Polq*-ΔPE cells were transduced with lentiviral sgRNAs targeting *Sam68, Polq*, or a nontargeting control, and repair profiles were established as before. We indeed found that while depletion of Sam68 or Polθ reduced TMEJ in wild-type cells, Sam68 depletion no longer altered the DSB repair profile in *Polq*-ΔPE cells, whereas Polθ depletion still affected TMEJ (Fig. 5F and G, and Supplementary Fig. S5C). These results demonstrate that inclusion of the PE in *Polq* transcripts underlies the reduced TMEJ activity observed upon Sam68 depletion. To test whether removal of the PE sequence also affects the IR-sensitivity of Sam68 deficient cells (Fig. 2G) we performed clonogenic survivals following IR using wild-type and *Polq*-ΔPE cells transduced with sgRNAs targeting *Sam68, Polq*, or a nontargeting control. Whereas depleting Sam68 in wild-type cells resulted in a mild sensitivity to IR (at higher doses), we found that depleting Sam68 in *Polq*-ΔPE cells no longer sensitizes cells to IR, suggesting that at least part of the sensitivity of *Sam68* knockout cells can be explained by reduced TMEJ activity (Supplementary Fig. S5D).

Finally, we followed up on one interesting earlier result: the observed ∼25% remaining *Polq* transcripts lacking the PE in *Sam68* knockout cells (Fig. 3E) is apparently insufficient to fully support TMEJ. In wild-type cells, ∼55% of *Polq* transcripts do not contain the PE. Therefore, we consider an approximately twofold reduction in correctly processed transcripts upon Sam68 depletion. These observations suggest that TMEJ is dosage-sensitive and predict that (i) cells heterozygous for a *Polq* deletion allele may exhibit reduced TMEJ activity, and (ii) Sam68 depletion would further impair TMEJ in such cells. To test these predictions, we generated *Polq* heterozygous (*Polq*^+/−^) cells and repeated the experiment described in Fig. 5E. Deletion of one *Polq* allele indeed reduced TMEJ-associated repair outcomes, revealing that TMEJ is sensitive to Polθ dosage, even in situations with very limited number of DSBs. The extent of TMEJ-outcome reduction approaches the level observed upon Sam68 loss, which could suggest that the drop in functional *Polq* transcripts under Sam68-depleted conditions is at least 50% (Fig. 5H and I). We surprisingly found that *Polq*^+/−^ cells were not hypersensitive to IR, suggesting that TMEJ is more affected in Sam68 depleted cells than in these *Polq*^+/−^ heterozygous cells, although we cannot formally exclude that the previous observed IR sensitivity of *Sam68* knockout cells results from additional DNA repair impairments (Supplementary Fig. S5E) [31, 32]. Furthermore, we found that Sam68 depletion in *Polq*^+/−^ cells further reduced TMEJ, approaching the phenotype observed in *Polq*^−/−^ cells (Fig. 5H and I). Together, our findings identify Sam68 as a critical regulator of *Polq* splicing required to express sufficient functional Polθ for efficient TMEJ (Fig. 5J).

## Discussion

In this study, we identified the RNA-binding protein Sam68 as a regulator of the intrinsically mutagenic DSB repair pathway TMEJ in mammalian cells. Specifically, Sam68 functions as a splicing modulator, inhibiting the inclusion of a PE in *Polq* mRNA that would otherwise lead to a premature termination and loss of Polθ expression (Fig. 5J). Importantly, deletion of this PE restores TMEJ activity in Sam68-deficient cells, demonstrating that altered *Polq* splicing is the principal mechanism underlying the observed repair defect. Our data further indicate that TMEJ is sensitive to Polθ dosage: already a partial reduction in functional *Polq* transcripts, either through Sam68 depletion or heterozygous *Polq* deletion, leads to measurable shifts in repair signatures at single break sites. Moreover, Sam68 depletion in a *Polq*^+/−^ background nearly phenocopies *Polq* loss, supporting a close relationship between Polθ levels and TMEJ output. This dosage dependence suggests that modest changes in *Polq* splicing can substantially influence DSB repair pathway engagement. Regulation at the RNA level may therefore provide a rapid and reversible mechanism to suppress or activate TMEJ in cells. Notably, analogous regulation has recently been reported for the Fanconi anemia pathway, in which the splicing factor CCAR1 was shown to suppress PE inclusion in *FANCA* pre-mRNA, suggesting that controlled alternative splicing may represent a more general mechanism for tuning DNA repair pathway activity [37, 38].

The precise timing of when *Polq* splicing regulation is critical remains unclear, leaving the temporal dynamics of this alternative splicing activation and repression currently elusive. PEs are mostly recognized as crucial regulators of development, with their inclusion tightly controlled at specific stages [39]. Therefore, one tempting hypothesis is the possibility that *Polq* splicing regulation via Sam68 is embedded within developmental programs. It is conceivable that, at distinct developmental stages, upregulation of Sam68 suppresses PE inclusion, thereby enhancing TMEJ activity. In agreement with this hypothesis, the protein expression landscape of early embryos reveals that Sam68 protein expression is upregulated during the early developmental stages (from zygote to blastocyst). In addition, Sam68 is part of translation regulation in the pre-implantation development of mouse embryos [40, 41]. It has also been shown that Sam68 is highly expressed in meiotic cells, where it controls an extensive splicing program essential for male sperm cells [42]. Notably, our RNA-seq analysis identified a meiotic factor (SIX6OS1) as the top Sam68-regulated splicing target. During early development and meiosis, cells experience replication-associated stress and programmed DNA breaks while maintaining strict genome integrity. Regulated PE inclusion may serve to control mutagenic TMEJ activity during these sensitive stages, with Sam68 enabling increased Polθ production when elevated repair capacity is required (e.g. to serve as a backup mechanism to resolve persistent or improperly processed meiotic recombination intermediates, thereby safeguarding chromosome segregation when HR fails).

In embryonic stem (ES) cells, increased inclusion of the PE could suppress TMEJ, potentially protecting pluripotent stem cells from precocious mutagenic repair that could compromise genomic integrity. Supporting this hypothesis, a previous study reported that mES cells express lower levels of Polθ and exhibit greater inclusion of the PE in *Polq* (stimulated by the ES cell specific RNA-binding protein DPPA5A) compared to their differentiated isogenic ES cells and MEFs, suggesting that Polθ expression may increase as cells differentiate [43, 44].

Post-translational modifications of Sam68 may further modulate its impact on *Polq* splicing and TMEJ. Sam68 is a substrate of the DNA damage kinase ATM, and ATM-dependent phosphorylation on conserved serine/threonine residues enhances its RNA binding affinity and splicing activity upon genotoxic stress, linking DNA damage signaling with RNA processing [45–47]. By contrast, Cdk1-mediated phosphorylation at defined threonine sites reduces Sam68’s alternative splicing activity, alters its RNA binding and cellular localization, and potentially attenuates its ability to regulate splicing of target genes [48]. These observations suggest that dynamic phosphorylation of Sam68 in response to cell cycle and DNA damage cues could influence *Polq* PE inclusion rates and, consequently, TMEJ capacity in different cellular states. Sam68 has also previously been implicated in the DNA damage response, primarily by regulating DNA damage-induced formation of poly(ADP-ribose) (PAR) chains through its interaction with PARP1 [31, 32]. While this could contribute to the mild IR-sensitivity we observe in Sam68 knockout cells, it is unlikely to directly contribute to the effect on TMEJ, as it was shown that PARP1 is not involved in this end-joining pathway [49].

Finally, Sam68 is expressed at high levels in several cancer types and has been implicated in oncogenic processes [50, 51]. Our results provide a mechanistic link between RNA splicing regulation and mutagenic DNA repair: increased Sam68 expression could reduce PE inclusion and elevate Polθ levels, thereby promoting TMEJ-dependent repair and enhancing the survival capability of fast-cycling cancer cells. Interestingly, it was previously shown that Sam68 is a negative prognostic factor in breast cancer and that breast cancer stem-like cells express high levels of Sam68 and the HR-protein Rad51, which help them to resist chemo- and radiotherapy by enhancing DNA damage repair [31]. Targeting both Sam68 and Rad51 in those cells induced synthetic lethality, similar to how inhibition of Polθ triggers cell-death in BRCA1-deficient tumors [7, 52–54]. Whether the regulation of *Polq* PE inclusion is part of the observed synthetic lethal interaction between Sam68 and Rad51 remains to be elucidated. Conversely, Sam68 has also been shown to promote TNFα-mediated apoptosis in BRCA2-deficient cells, thereby contributing to the elimination of genomically unstable cells [55]. Together, these findings suggest that the function of Sam68 is highly context dependent, with distinct roles during the early cellular response to genomic instability and in the maintenance and survival of established tumors. Further work will be required to determine whether *Polq* splicing status correlates with Polθ expression or mutational signatures in human tumors and whether Sam68’s effects on splicing contribute to therapy response in HR-deficient cancers.

## Supplementary Material

gkag783_Supplemental_Files

## Data Availability

The raw targeted sequencing data generated in this study have been deposited in the NCBI SRA database (https://www.ncbi.nlm.nih.gov/sra) under accession code PRJNA1436449. A description of the sequence files can be found in Supplementary Table 4. The RNA sequencing data generated in this study has been deposited in the GEO database (https://www.ncbi.nlm.nih.gov/geo/) under accession number GSE324893.
