# Supplementary material for: Sam68 stimulates polymerase theta-mediated end-joining by suppressing poison exon inclusion in Polq mRNA
Source: Nucleic Acids Res. 2026 Aug 11;54(15):gkag783. doi: 10.1093/nar/gkag783 (PMC13458401; doi:10.1093/nar/gkag783)
Supplement: Supplementary file 1 [file gkag783_supplemental_files.zip › 20260707_Wezel-etal_2026_Supplementary-file.pdf]

**Supplementary file**

# Sam68 stimulates polymerase Theta-Mediated End-Joining by suppressing poison exon inclusion in *Polq* mRNA

Marloes D. van Wezel[1], Hanneke Kool[1], Diana van den Heuvel[1], Robin van Schendel[1], Marco Barazas[1], Martijn S. Luijsterburg[1], Marcel Tijsterman[1,2*], Joost Schimmel[1*]

[1]Department of Human Genetics, Leiden University Medical Center, The Netherlands.

[2]Institute of Biology Leiden, Leiden University, The Netherlands.

[*]co-corresponding authors

j.schimmel@lumc.nl

m.tijsterman@lumc.nl

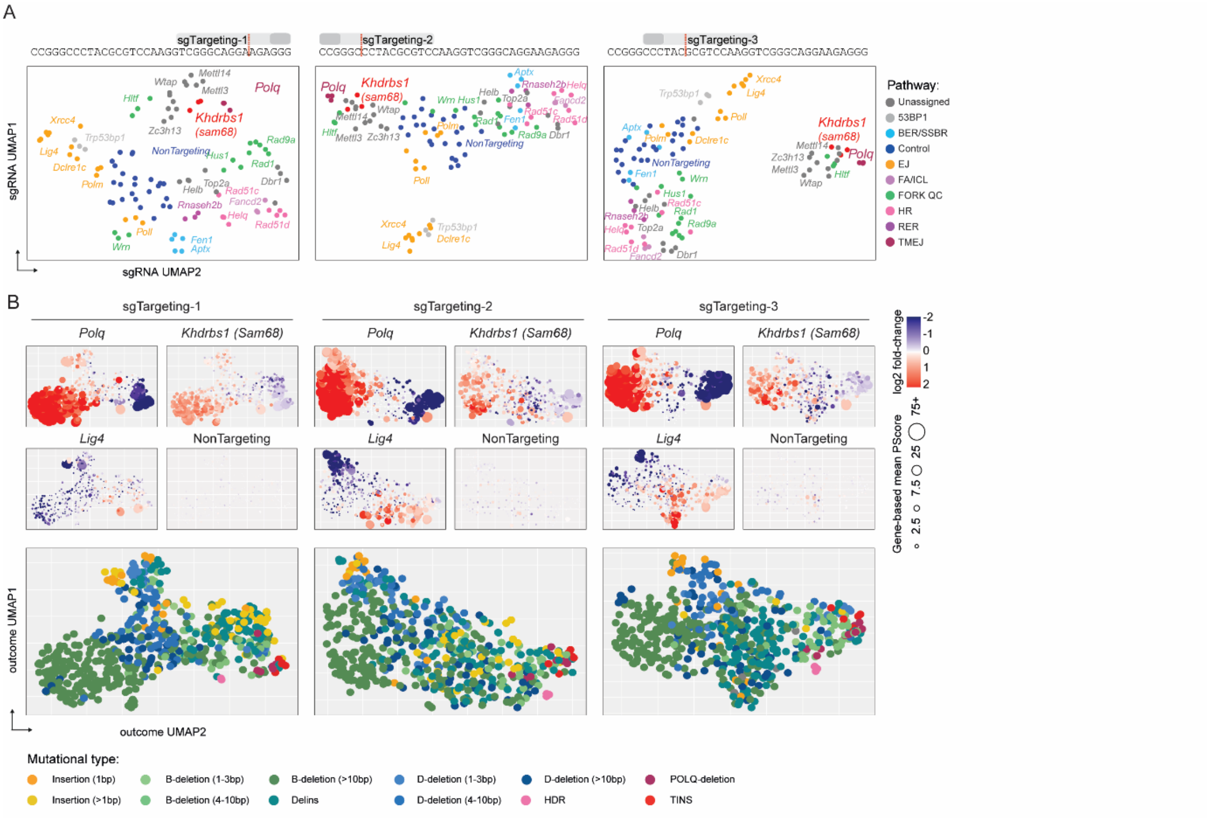


**Figure S1. sgRNA and outcome UMAPs for the three target-sites used in the MUSIC setup**

(A) Using custom SIQ software (29), the deconvolution of a subset of the published MUSIC dataset (17) produces a UMAP projection of the top outlier sgRNAs for deviation in mutational signatures at three different CRISPR/Cas9 target sites (sgLibrary-1, -2 and -3). Each dot represents an individual sgRNA, positioned based on the similarity of its repair outcome distribution to that of other outliers and non-targeting sgRNAs. Colors represent the DNA repair pathway associated with each sgRNA. One label is displayed per gene in the UMAP. (B) (top) UMAP projection of mutational outcomes for the three different CRISPR/Cas9 target sites. The log2 fold-change in repair outcomes for, *Polq*, *Khdrsb1* (*Sam68)* and *Lig4* sgRNAs (average of 3 sgRNAs per gene), and one NonTargeting sgRNA is shown; dots are sized based on Pscore. (bottom) corresponding UMAPs for sgLibrary-1, -2 and -3 with dots color-coded by mutational type. Insertion (1bp) contained insertions of one base pair; insertions (>1bp) contains all insertions larger than one base pair; POLQ-deletion contained the three most common deletions that were verified to be POLQ-dependent; deletions are further annotated based on whether DNA loss occurs at one or both DSB ends (directional (D) versus bi-directional (B) deletions; deletion (1-3bp) contained deletions of 1-3 bp in size; deletion (4-10bp) contained deletions of 4-10 bp in size; deletion (>10bp) contained deletions of at least 11 bp in size; HDR is representing a specific event in which the targeting backbone serves as a repair template; delins contained deletions associated with insertions; tins (templated insertions) are a subclass of delins in which the insertion exhibits a sufficiently long match (>6bp) to nearby DNA sequence at the break junction.

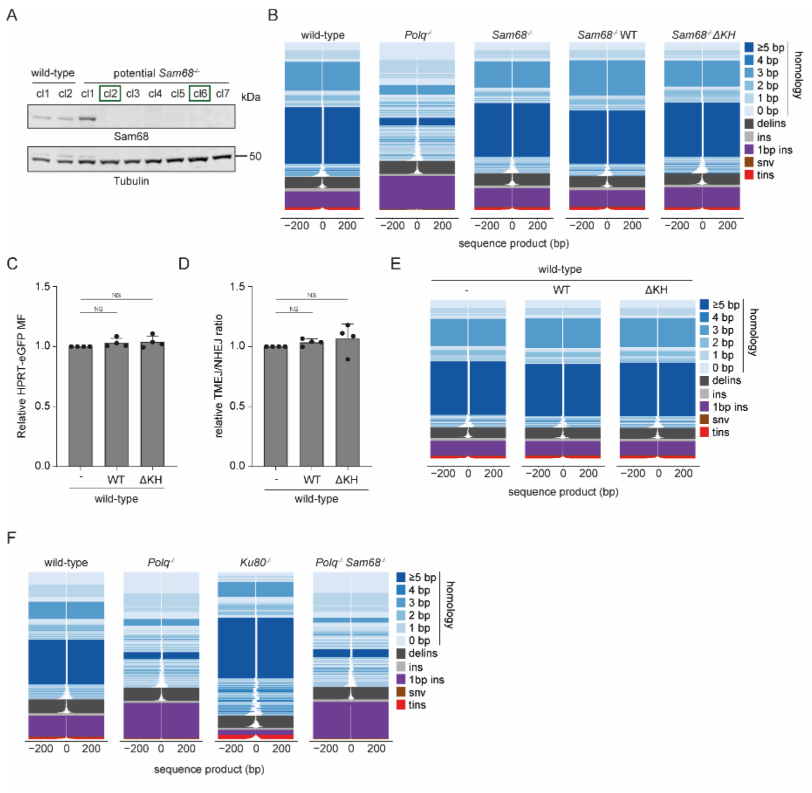


**Figure S2. *Sam68* knockout validation and complementation**

(A) Western blot of two wild-type and seven potential *Sam68* knockout clones. Clone 2 and Clone 6 are used in this study. Tubulin serves as a loading control. Cl, clone. (B) Mutational signatures at the Cas9-induced DSB for the indicated mES cell lines transfected with Cas9-WT and the sgRNA targeting *HPRT* exon 3. Cells were collected for DNA extraction and used for short-read targeted sequencing around the break site. Data are represented as tornado plots where the height of each bar reflects the contribution of each outcome to the total amount of reads within the condition. Data are plotted relative to the Cas9-WT break site (0) and sorted by mutational type. Mutation types are color-coded. The degree of blue coloring reflects the extent of microhomology present at the deletion junction. Data are from four independent experiments (n = 4). delins, deletion insertion; snv, single nucleotide variant; ins, insertion; tins, templated insertions; bp, base pair. (C) Relative *HPRT-eGFP* mutation frequency (MF) in the indicated mES cell lines transfected with Cas9-WT and the sgRNA targeting *HPRT* exon 3. Data are shown as mean ± SD (n = 4), with values expressed as a fraction of the mutation frequency observed in wild-type cells (set to 1). Statistical significance was determined using one-way ANOVA followed by Dunnett's multiple comparisons test; ns, not significant. (D) Quantification of TMEJ/NHEJ mutagenic events obtained by short-read targeted sequencing. The ratio of TMEJ/NHEJ mutagenic events in wild-type cells is set to 1. Data are shown as mean ± SD (n = 4). Ratio-based measures were log-transformed prior to analysis. Statistical significance was determined using one-way ANOVA followed by Dunnett's multiple comparisons test; ns, not significant. (E) Similar to (B), using indicated genotypes. Data are from four independent experiments. (F) Similar to (B, E), using indicated genotypes. Data are from three or six independent experiments.

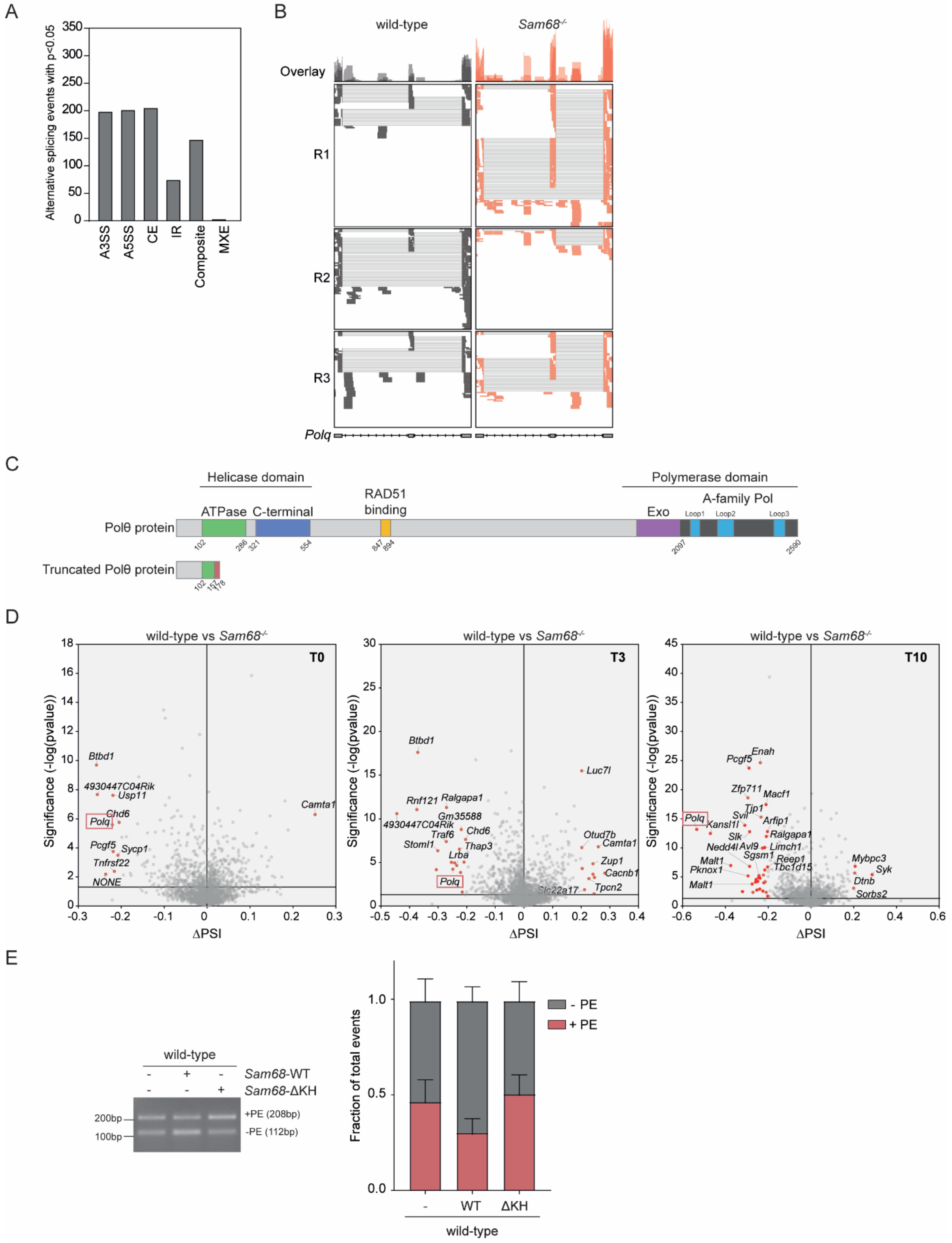


**Figure S3. RNA-seq data from wild-type and *Sam68* knockout mES cells**

(A) Absolute number of alternative splicing events altered in wild-type versus *Sam68*$^{-/-}$ cells, as identified using JUM software (27). Splicing event types are indicated as follows: A3SS (alternative 3' splice site), A5SS (alternative 5' splice site), CE (cassette exon), IR (intron retention), Composite (inseparable combinations of conventional

patterns), and MXE (mutually exclusive exons). (B) Sashimi plots showing RNA-seq read coverage usage for the poison exon event in the *Polq* transcript for wild-type and *Sam68* knockouts. Data are presented for three biological replicates (R1, replicate 1; R2, replicate 2; R3, replicate 3) for each cell line. (C) Schematic representation of the full-length Polθ and the truncated Polθ. Inclusion of the poison exon introduces a premature stop codon resulting in truncated Polθ (D) Volcano plots of CE-events from published data where wild-type and *Sam68*$^{-/-}$ mES cells were allowed to spontaneously differentiate to tissue-like spheroids during which RNA was isolated at three different time-points (day 0, 3 and 10) (28). Each dot represents a cassette exon event, with the x-axis showing ΔPSI (the difference in proportion of cassette exon usage between the two groups) and the y-axis representing significance ($-\log_{10}$(p-value)). Positive ΔPSI values indicate greater inclusion of the cassette exon in the wild-type cells, while negative values indicate greater inclusion in the *Sam68* knockout cells. Events that are statistically significant with a strong ($\Delta PSI < -0.2$ or $\Delta PSI > 0.2$) effect are shown in red. All other events are shown in grey. Gene associations were determined by JUM-analyses (27). (E) The ratio of poison exon inclusion/exclusion in *Polq* was determined in the indicated mES cell lines using RT-PCR followed by agarose analysis, and short-read targeted sequencing of the cDNA. Data are shown as mean ± SD (n = 2).

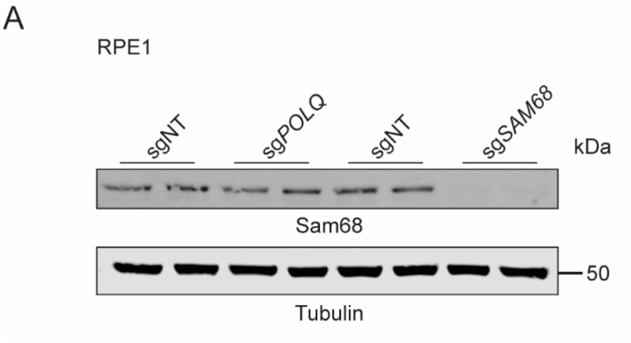


**Figure S4. Sam68 depletion in RPE1 polyclonal cells**

(A) Western blot of RPE1 cells showing depletion of Sam68 after lentiviral transduction of two independent sgRNAs targeting *Sam68*. Tubulin serves as a loading control.

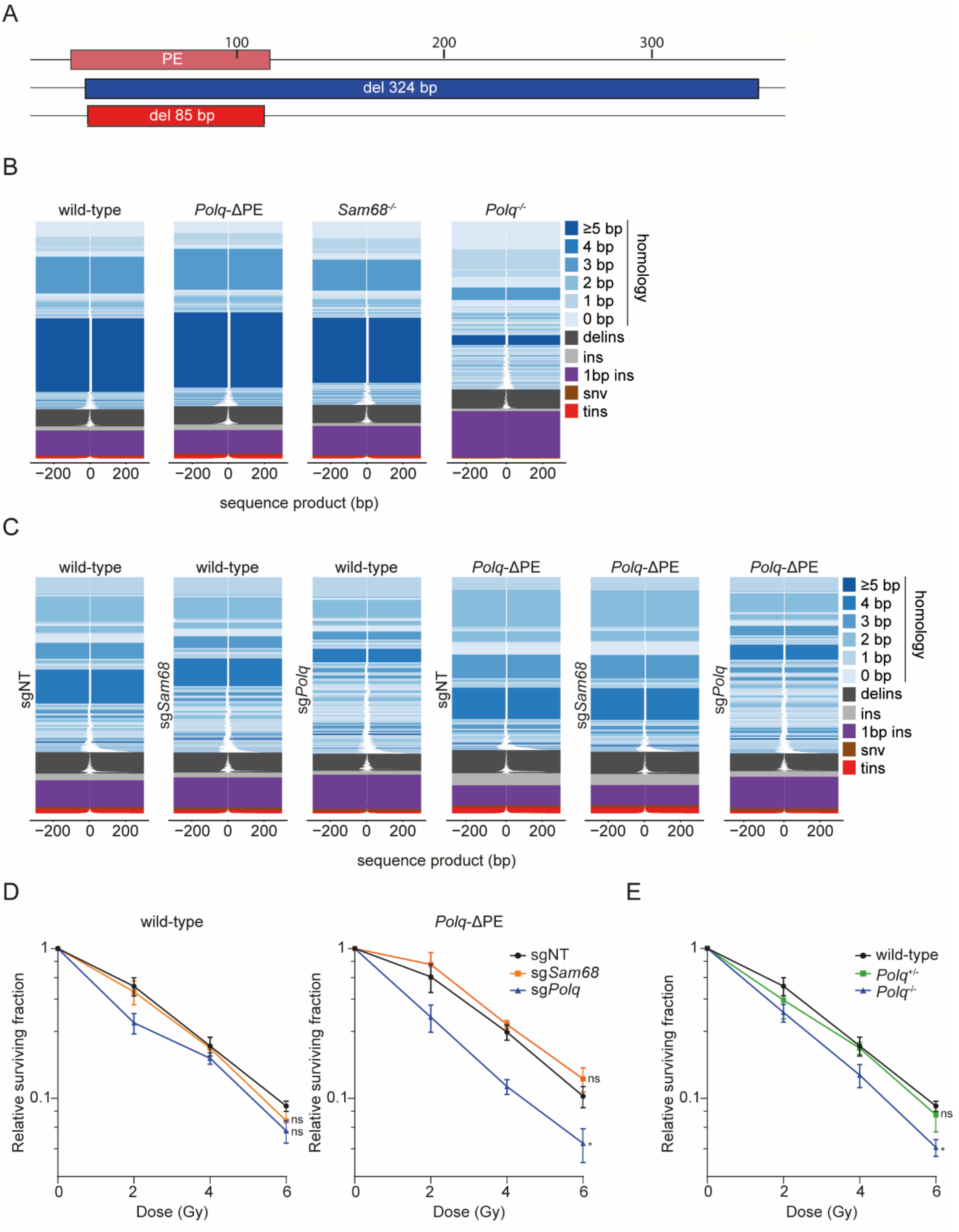


**Figure S5. CRISPR/Cas9-induced mutational signatures in *Polq*-ΔPE mES cells**

(A) Schematic illustration of both alleles in the poison exon knock-out clone. Sequencing data from the knockout clone was obtained using Sanger sequencing. (B) Mutational signatures at the Cas9-induced DSB for the indicated mES cell lines transfected with Cas9-WT and the sgRNA targeting *HPRT* exon 3. Cells were collected for DNA extraction and used for short-read targeted sequencing around the break site. Data are represented as tornado

plots where the height of each bar reflects the contribution of each outcome to the total amount of reads within the condition. Data are plotted relative to the Cas9-WT break site (0) and sorted by mutational type. Mutation types are color-coded. The degree of blue coloring reflects the extent of microhomology present at the deletion junction. Data are from three independent experiments (n = 3). delins, deletion insertion; snv, single nucleotide variant; ins, insertion; tins, templated insertions; bp, base pair. (C) Mutational signatures at the Cas9-induced DSB for the indicated Cas9-expressing mES cell-lines transduced with the sgRNA targeting the library backbone. Cells were collected for DNA extraction and used for short-read targeted sequencing around the break site. Data are represented as tornado plots where the height of each bar reflects the contribution of each outcome to the total amount of reads within the condition. Data are plotted relative to the Cas9-WT break site (0) and sorted by mutational type. Mutation types are color-coded. The degree of blue coloring reflects the extent of microhomology present at the deletion junction (n = 4). delins, deletion insertion; snv, single nucleotide variant; ins, insertion; tins, templated insertions; bp, base pair. (D) Clonogenic survival of wild-type and *Polq*-ΔPE mouse ES cell lines expressing a non-targeting sgRNA or sgRNAs targeting *Sam68* or *Polq*, following exposure to varying doses of ionizing radiation. Data are presented as mean ± SEM (n = 6, with three independent experiments using two sgRNAs per target gene). (E) Clonogenic survival of wild-type, *Polq*$^{+}$/$^{-}$, and *Polq*$^{-}$/$^{-}$ mouse ES cell lines following exposure to varying doses of ionizing radiation. Data are presented as mean ± SEM (n = 6, with three independent experiments using two clones per genotype).
